# A Systematic Review and Meta-Analysis of Wound Complications after a Caesarean Section in Obese Women

**DOI:** 10.3390/jcm10040675

**Published:** 2021-02-10

**Authors:** Aneta Słabuszewska-Jóźwiak, Jacek Krzysztof Szymański, Łukasz Jóźwiak, Beata Sarecka-Hujar

**Affiliations:** 1 First Department of Obstetrics and Gynaecology, Centre of Postgraduate Medical Education, ul. Żelazna 90 s, 01-004 Warsaw, Poland; jkszymanski2@gmail.com; 2 Department of Obstetrics and Gynaecology, St. Sofia Hospital, ul. Żelazna 90, 01-004 Warsaw, Poland; luk2jozwiak@gmail.com; 3 Department of Basic Biomedical Science, Faculty of Pharmaceutical Sciences in Sosnowiec, Medical University of Silesia, ul. Kasztanowa 3, 41-200 Sosnowiec, Poland; beatasarecka@poczta.onet.pl

**Keywords:** caesarean section, wound complication, wound morbidity, wound infection, surgical-site infection

## Abstract

(1) Background: Caesarean sections in obese patients are associated with an increased risk of surgical wound complications, including hematomas, seromas, abscesses, dehiscence, and surgical site infections. The aim of the present study is to perform a meta-analysis and systematic review of the current literature focusing on the strategies available to decrease wound complications in this population. (2) Methods: We reviewed the data available from the PubMed and the Science Direct databases concerning wound complications after caesarean sections in obese women. The following key words were used: “caesarean section”, “cesarean section”, “wound complication”, “wound morbidity”, and “wound infection”. A total of 540 papers were retrieved, 40 of which were selected for the final systematic review and whereas 21 articles provided data for meta-analysis. (3) Results: The conducted meta-analyses revealed that the use of prophylactic drainage does not increase the risk of wound complications in obese women after a caesarean sections (pooled OR = 1.32; 95% CI 0.64–2.70, *p* = 0.45) and that vertical skin incisions increase wound complications (pooled OR = 2.48; 95% CI 1.85–3.32, *p* < 0.01) in obese women, including extremely obese women. (4) Conclusions: Subcutaneous drainage does not reduce the risk of a wound complications, wound infections, and fever in obese women after caesarean sections. Negative prophylactic pressure wound therapy (NPWT) may reduce the risk of surgical site infections. The evidence of using a prophylactic dose of an antibiotic before the caesarean section is still lacking.

## 1. Introduction

Obesity is a chronic disease that leads to the development of metabolic disorders and cardiovascular complications and currently poses a challenge for healthcare systems around the world [1]. According to the World Health Organisation (WHO), the problem of obesity is reaching epidemic proportions, in both developed as well as developing countries. In 2016, 15% of women over 18 years of age were either overweight or obese [2]. This problem also affects 1/3 of women of reproductive age [3], including 13% of pregnant women [4]. The rising prevalence of weight gain during pregnancy is associated with the occurrence of a greater number of complications during pregnancy, childbirth, or the postpartum period [5]. Research confirms that being overweight may also increase the rate of complications in both pregnant women and newborn babies [6]. Obese women develop arterial hypertension [7] and diabetes more often in pregnancy and undergo caesarean sections significantly more often [8], which means that they are diagnosed with postoperative surgical wound healing disorders more frequently [9].

Postoperative complications include: superficial infections, dehiscence, or the presence of a fluid reservoir (seroma and hematoma) at the wound site. The above symptoms concern 3 to 15% of women after a caesarean section [10,11] and often result in prolonged hospitalization, antibiotic therapy, thus leading to increased postpartum care costs. A superficial infection is part of a surgical site infection (SSI) which, according to the Centers for Disease Control and Prevention (CDC), is an infection that occurs within 30 days of the performed surgical procedure. The risk factors for the above complications include young age at childbirth, smoking, obesity, arterial hypertension, diabetes, chorioamnionitis, increased intrapartum blood loss, prolonged ruptured of membrane, emergency caesarean section and subsequent surgical delivery, use of suboptimal antibiotic prophylaxis, improper preparation of the surgical field, extended duration of the surgical procedure, and the employed caesarean section technique, including that of the incision and of the suturing of the skin [12,13,14,15].

The aim of the study is to review the current literature in order to determine the factors influencing the healing of postoperative wounds in obese women undergoing a caesarean section.

## 2. Materials and Methods

This systematic review was conducted in accordance with the Preferred Reporting Items for Systematic Reviews and Meta-Analyses (PRISMA) guideline [16].

### 2.1. Search Strategy

We searched the following databases: PubMed, Science Direct Web of Science as well as Google Scholar for relevant papers published from 2010 to 2020 (last search in October 2020). The small number of studies on subcutaneous drainage in this period contributed to an extended search strategy in this field for the years 2000–2020. The following key words were used: “caesarean section,” “cesarean section,” “wound complication,” “wound morbidity,” “wound infection.” Two authors (A.S.-J., B.S.-H.) independently reviewed the search results to identify relevant studies and disagreements between the reviewers were arbitrated. The study titles and abstracts were screened according to the following inclusion/exclusion criteria. The references cited in the found articles were also searched in order to identify other published articles on the topic. The selected relevant studies selected were classified according to the population, intervention, comparison, and outcome (PICO) framework in order to identify the relevant research questions meeting the following selection criteria:

Population: obese women;

Intervention: caesarean section;

Comparison: women with normal weight vs. overweight women;

Outcomes: wound infection, wound complications, and wound morbidity.

Separate searches were performed for each of the topics covered in this review followed by the deletion of any duplicates. At the first step of elimination, the studies that were clearly irrelevant based on their title were removed. Next, the remaining abstracts were reviewed and those that were irrelevant to the topic were excluded. The remaining papers were comprehensively read to determine whether they contain the relevant information.

### 2.2. Inclusion and Exclusion Criteria

Retrospective, prospective control, and randomized cohort studies reporting wound complication after elective, emergency or intrapartum caesarean sections in obese women, who were defined as women with a body mass index (BMI) of ≥30 kg/m^2^ before pregnancy or at the time of delivery, were considered. Studies available in English with a publication date ranging from 2010 to 2020 and available in English describing the placement of a skin incision, wound dressing, or skin closure was included into the final analysis.

The exclusion criteria were a caesarean section performed before 23 weeks of pregnancy and participants aged under 18 or over 45 years. Reviews, commentaries, and case reports were also excluded.

### 2.3. Selection Process and Result Codification

The search, selection, and analysis of the studies found during the literature search were conducted independently by two team members. The selection was based on reading the title and abstract, followed by the full text of the paper and, finally, a reverse search in the selected studies. Outcomes were considered clearly defined if the authors provided an adequate level of detail about of criteria. Disagreements were resolved through arbitration and discussion with other authors. For the meta-analysis, we selected studies that used the same measuring instrument and provided the data necessary for its execution.

### 2.4. Critical Reading and Level of Evidence

The studies included in the research were reviewed critically for bias analysis using the Strengthening the Reporting of Observational Studies in Epidemiology (STROBE) checklist [17]. The selected studies were assigned a methodological quality grade according to the levels of evidence and degrees of recommendation proposed by the Jadad scale [18] (Supplementary Table S1).

### 2.5. Statistical Analysis

Statistical analyses were performed using the Review Manager software (RevMan version 5.4 Cochrane, London, UK) and MedCalc software (version 19.5.3.; MedCalc Software Ltd., Ostend, Belgium). The pooled odds ratio (OR) along with a 95% confidence interval (CI) for the following comparisons: women with a drain vs. women without a drain, women with vertical skin incision vs. women with transverse skin incision, women with NPWT vs. women with standard therapy in terms of wound complications, infections, as well as fever were calculated. The results of the heterogeneity between studies, i.e., the I^2^ test at level of 50%, allowed to use between the random effects model (REM) or the fixed effects model (FEM). I^2^ expresses the proportion of dispersion due to heterogeneity and I^2^ at 25%, 50%, and 75% was suggested as low, intermediate, and high inconsistency. The publication bias was evaluated both visually with inspection of funnel plots and by performing Egger’s test as well as Begg’s test; however according to the recommendations, at least 10 studies must be included to conduct funnel plot asymmetry tests so as to maintain sufficient power for distinguishing the chance from real asymmetry [19,20]. In addition, the sensitivity analyses were performed by omitting each included study at a time (the leave-one-out method) to reflect the influence of the individual data set to the pooled OR and in consequence to assess whether the results remained stable and reliable. The results of the carried out meta-analyses were summarized in tables and illustrated using forest plots.

## 3. Results

### 3.1. Study Selection Process

A total of 540 articles were first selected. For the initial screening, 30 duplicates were identified and removed, leaving 510 articles. The titles and abstracts were then assessed by two reviewers, ending with the inclusion of 94 articles. The full texts were then retrieved for those citations that were considered potentially relevant and assessed in terms of their eligibility by the two reviewers. Of these 94 articles, 34 were excluded. The most common reasons for the exclusions were case reports and a language other than English. The reference lists of the included studies were hand searched by the first author. Separate searches were performed for each of the topics covered in this review. The first elimination step involved removing, the studies that were clearly irrelevant based on their title followed by excluding any duplicates. Next, the remaining abstracts were reviewed and the irrelevant ones in terms of the topic were removed. The full text of the paper was read for all the remaining papers in order to determine whether they contained the relevant information.

**Figure 1:**
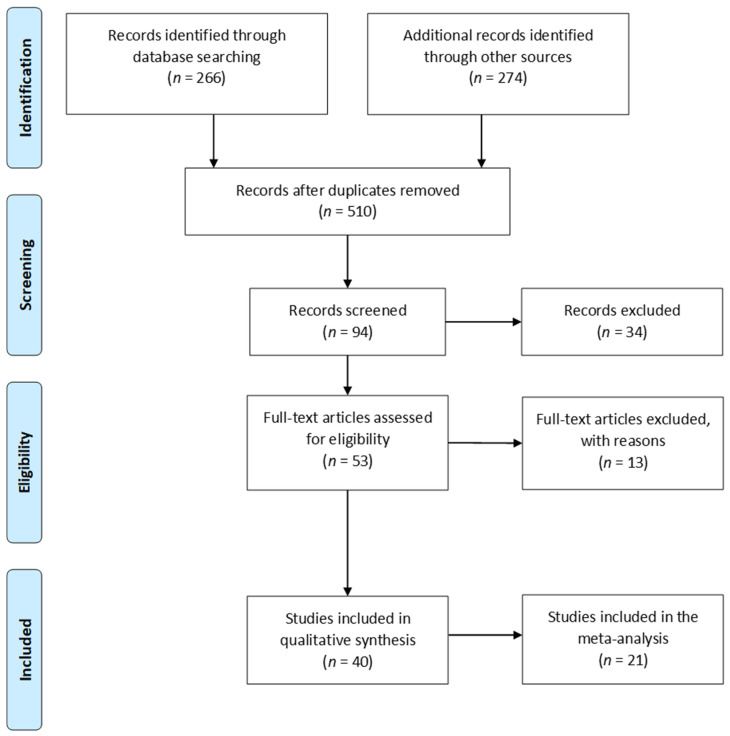
Flow chart presenting the process of searching for eligible articles according to PRISMA guidelines.

Eventually, a total of 40 relevant articles [10,11,21,22,23,24,25,26,27,28,29,30,31,32,33,34,35,36,37,38,39,40,41,42,43,44,45,46,47,48,49,50,51,52,53,54,55,56,57,58] were included in the current systematic literature review whereas 21 articles provided data for meta-analysis. Six studies regarding skin incision [24,26,27,31,33,42] and 9 studies regarding drainage [21,22,23,24,25,27,32,35,46] were performed in meta-analysis. The frequency of surgical site infection (SSI) in obese women with negative pressure wound therapy (NPWT) in comparison to women with standard therapy was based on 6 studies [36,37,39,43,45,47]. A summary of the search process is illustrated in Figure 1, as recommended by the PRISMA guidelines [16].

### 3.2. Characteristics of Included Studies

The studies included in the systematic review are retrospective cohort studies or a secondary analysis of observational cohort studies [21,22,23,24,25,26,27,28,29,30,31,32,33,34,35,36,37,38,39,40,41,42,43,44,45,46,47,48,49,50,51,52,53,54,55,56,57,58] and concern factors that have a potential impact on wound healing in obese patients (BMI ≥ he kg/m^2^) subjected to elective, unplanned, or intrapartum caesarean section. The factors influencing the proper healing processes include concomitant diseases such as arterial hypertension, diabetes, and smoking, as well as the premature rupture of membranes, an infection of the amniotic sac, the method of incision (vertical or transverse), and skin suturing (staplers, intradermal sutures, running, or intermittent sutures), and the use of prophylactic vac dressings. When assessing the effects of the surgical procedure, the authors reported complications including: wound infections, endometritis, the presence of a hematoma or serous exudate, and the discontinuation (dehiscence) of the wound edges that are the cause of rehospitalization, which was analyzed in six studies [24,26,27,31,33,42]. In evaluating the effectiveness of vac dressings, the primary goal was to assess the presence of a superficial incisional surgical site infection (SSI). Eleven studies assessed the use of cefazolin in perioperative prophylaxis [48,49,50,51,52,53,54,55,56,57,58], six of which were prospective [48,52,54,55,56,57,58], and two were randomized and double-blind [51,53].

The research was carried out in ten different countries, on four continents, mainly in North America. Twenty-three studies were conducted in the United States [10,11,28,29,31,32,33,34,36,39,40,42,43,46,47,48,50,51,52,53,55,56,57], and the remaining studies were carried out in Egypt [22,30,32,41], Denmark [44], India [35], Japan [25], Canada [54], Australia [21,58], and New Zealand [49]. Moreover, one study concerned women living in England or Ireland [38] and one in Scotland [46]. Most of the studies included both elective and unplanned caesarean sections (*n* = 14) with the exception of three studies that included emergency caesarean sections as the exclusion criterion [29,30,31]. In five cases, the surgical procedure was generally referred to as a caesarean section [10,26,34,38,41].

The degree of obesity was defined in accordance with the WHO definition across all studies, distinguishing obesity of the first (BMI 30.0–34.9 kg/m^2^), the second (BMI 35.0–39.9 kg/m^2^), and the third stage (BMI ≥ 40 kg/m^2^) [2]. Eight of the analyzed studies only concerned complications in women with morbid obesity, that is, with a BMI of distinguish^2^ [24,31,33,36,40,42,43,45].

Table 1 and Table 2 describe the full characteristics of the studies, including the place, the type of study, the size of the group, and the primary goals and results.

### 3.3. Synthesis of Results

#### 3.3.1. Relationship between Preoperative Factors and Wound Complications

Hypertension, like diabetes, is a disease that often coexists with obesity. Seven randomized trials investigate the effect of these diseases on wound healing [11,24,26,27,28,34,40], and take into account the premature rupture of the membranes, concomitant infections of the membranes or smoking. An American retrospective study found that 1 in 3 women who are morbidly obese will develop complications in the surgical wound, and smoking increases the risk of these complications by more than double (RR 2.7 [95% CI 1.08–6.54] *p* = 0.03) [24]. Another study confirmed that smoking and longitudinal skin incisions significantly increase the risk of infections and wound dehiscence after a caesarean section [40]. However, Temming and colleagues, in a study involving 1082 patients with a BMI of >30 did not confirm that obesity, smoking, diabetes, and chorioamnionitis had a significant impact on the risk of complications in the postoperative wound if the surgery was performed according to evidence-based medicine, that is, prophylactic antibiotics administered up to 60 min before the skin incision, the skin washed with an alcohol solution of chlorhexidine, the subcutaneous tissue sutured if its thickness is greater than 2 cm, and the skin sutured with an intradermal stitch. In this case, the only significant risk factor for wound disorders (27.5% vs. 16.1%, RR 1.71 [95% CI 1.12–247]) and the occurrence of SSIs (6.9% vs. 1.6%, RR 3.74 [95% CI 1.18–11.92]) is the fact of an emergency caesarean section [11]. Other authors showed no significant influence of diabetes, hypertension, the premature outflow of amniotic fluid or inflammation of the membranes on the increased incidence of postoperative wound complications in obese women [24,26,27,28,34].

#### 3.3.2. Antibiotic Prophylaxis

The estimation of the optimal prophylactic dose of an antibiotic reaching a concentration higher than the minimum inhibitory dose (minimum inhibitory concentration)—both in the blood and in the adipose tissue—reducing the risk of postoperative wound infection, remains the subject of many studies [48,49,50,51,52,53,54,55,56,57,58]. In a 2011 study, Pevzner and colleagues questioned the effectiveness of a prophylactic dose of 2 g of cefazolin in women with varying degrees of obesity [48]. This was confirmed by Swank and colleagues, who showed that the minimal inhibitory concentration (MIC) was not reached in adipose tissue in relation to Gram-negative bacteria at the time of skin incision in 20% of obese women (BMI 30–40 kg/m^2^) and 44% of morbidly obese women after the use of cefazolin in a dose of 2 g. Increasing the dose to 3 g resulted in all the obese and 71% of the morbidly obese women reaching MIC ≥8 µg/mL [52]. In a randomized Australian cohort study involving 2231 women, increasing the dose of cefazolin to 3 g in women with a BMI of ≥30 kg/m^2^ resulted in a significant reduction in the occurrence of SSIs (OR 0.309, *p* < 0.001) [59]. In a randomized, double-blind study conducted among pregnant women in labor with a BMI of above 30, increasing the dose of cefazolin to 3 g did not significantly increase its concentration in adipose tissue and did not show significantly greater protection against the *Staphylococcus* species compared to the group receiving a dose of 2 g (61% vs. 72%, *p* = 0.35) [53]. In another retrospective US cohort study involving 335 obese women, increasing the prophylactic dose also did not reduce the incidence of SSIs [50]. In a randomized, double-blind study, Young et al. showed that although the concentration of the antibiotic in both blood serum and pregnant adipose tissue is dependent on the dose of the drug and the body weight, the use of both 2 g and 3 g of cefazolin is the optimal protection in the ratio of Gram-positive and Gram-negative bacteria [51]. Similar conclusions were provided by the study conducted by Groff and colleagues, which showed that the intravenous administration of 2 g of cefazolin fully protects against postoperative wound infections in both obese and normal-weight women. Moreover, this dose in both groups protects the newborn against an infection with the *Streptococcus group B* (GBS) and S. aureus [55]. Another American study investigating the penetration of an antibiotic into adipose tissue showed that the administration of 2 g of cefazolin 30–60 min before the skin incision reached concentrations above the minimum inhibitory concentration in both overweight and obese women if the procedure lasts less than 90 min (the probability of target attainment (PTA) in adipose tissue for 2 g of cefazoline was 92.4%, and 94.7% for 3 g of cefazoline). If the duration of the surgery exceeds 2 h, the PTA for 2 g of cefazoline was 86.8%, suggesting the need for another dose of antibiotic [56]. Eley et al. also confirmed that another prophylactic dose in the case of routine, uncomplicated patients is not needed [58]. However, Guper et al. showed that both the total body weight (TBW) and the BMI had no effect on the concentration of cefazolin in adipose tissue [56]. On the other hand, Kram and colleagues, in a study involving 84 patients with obesity of at least grade 1, found that its mean concentration in adipose tissue is still below the MIC of 8 mg/g (*p* < 0.03) regardless of the antibiotic dose used [57]. The results of the analysis are presented in Table 3. 

#### 3.3.3. Skin Incisions

An incision of the skin and related complications within the postoperative wound were the subject of six retrospective randomized studies in which the incidence of complications following a transverse or vertical skin incision was analyzed. Most authors take into account the risk of wound infection and its dehiscence, as well as the presence of fluid collections (seroma and hematoma) resulting in rehospitalization. In a US cohort study of morbidly obese pregnant women, the risk of postoperative wound complications following a vertical abdominal incision more than doubled (OR 2.2 (1.18–4.27)) [24]. Another study involving 242 women with a BMI of ≥30 kg/m^2^ indicated that obesity significantly increases the risk of complications within a postoperative wound, although the method of skin incision had no effect on the incidence of this complication [26]. Smid et al., in an analysis of 2411 pregnant women, showed that those with morbid obesity (BMI > 45 kg/m^2^) are at increased risk of endometritis (AOR 1.26; 95% CI 1.07–1.49) and wound infections (AOR 3.77; 95% CI 2.60–5.46) compared to women with normal body weight. Moreover, he showed that infections accompany vertical incisions of the skin significantly more often (*p* = 0.02) than transverse incisions [31]. Thornburg et al. assessed that vertical incisions increase the risk of wound complications by more than seven-fold. There is an increased risk of infection (OR 5.16; 95% CI 2.3–11.8) and wound dehiscence (OR 10.7; 95% CI 4.0–29.2) in obese women, regardless of the degree of their obesity [27]. In the study from US, the authors showed a significantly lower incidence of complications within the wound (including infection, seroma, hematoma, and fascial dehiscence) in the case of vertical incisions of the skin (OR 0.32; 95% CI 0.17–0.62) [42]. The results are presented in Table 4.

**Figure 2:**
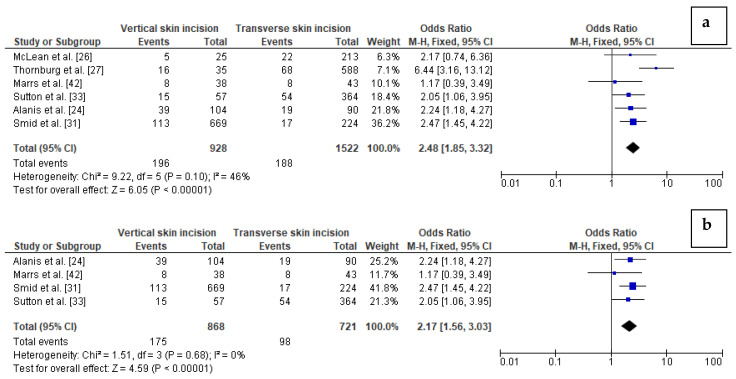
Forest plots for: (**a**) wound complications in the group of post-caesarean obese women with vertical skin incision and in women with transverse skin incision; (**b**) wound complications in the group of post-caesarean women with BMI ≥40 kg/m^2^ and vertical skin incision compared to women with transverse skin incision. M.-H.—Mantel-Haenszel; CI—confidence interval; I^2^—heterogeneity; df—degrees of freedom.

The impact of the type of skin incision on the risk of wound complication in obese women was assessed based on six studies with a total number of 928 women with vertical skin incision and 1522 obese women with transverse skin incision. Pooling the data together, we observed that wound complications were present in 21% of obese women with vertical incision but only in 12% of obese women with transverse incision. Studies were mildly moderately heterogeneous (I^2^ 46% with *p* = 0.10) thus the FEM method was used to calculate the pooled OR. Wound complications were found to be significantly more frequent in women with vertical skin incision than in women with transverse skin incision (21% vs. 12%). The value of pooled OR equals to 2.48 (95%CI 1.85–3.32, *p* < 0.01) indicates that the odd of wound complication in obese women after a caesarean section is almost 2.5-fold higher in the case of vertical skin incisions (Figure 2). No publication bias was found for this analysis [Egger’s test (*p* = 0.633); Begg’s test (*p* = 0.348)] and sensitivity analysis demonstrated that the results were stable and reliable.

In addition, we performed subgroup analysis based on four studies considering women with BMI ≥ 40 kg/m^2^, including 868 women with vertical skin incision and 721 women with transverse skin incision in total. There was no heterogeneity between the studies (I^2^ 0%). The analysis revealed that wound complications were again significantly more frequent in women with extreme obesity and vertical skin incision than in extremely obese women with transverse skin incision (20% vs. 14%, respectively). The results showed that the odd of wound complication in women with BMI ≥40 kg/m^2^ after caesarean section is almost 2.2-fold higher in the case of vertical skin incision than transverse skin incision (OR = 2.17 (95%CI 1.56–3.03, *p* < 0.01) (Figure 2). However, in this analysis publication bias was observed [Egger’s test (*p* = 0.008); Begg’s test (*p* = 0.041)]. In turn, sensitivity analysis showed that the results of OR calculation were stable.

#### 3.3.4. Skin and Subcutaneous Tissue Closure

Four randomized trials compared the effectiveness of stitching skin with staplers, single sutures, and intradermal sutures. An Egyptian study conducted among 130 women diagnosed with obesity whose skin was closed with an intradermal suture showed a significantly higher risk of SSIs and postoperative wound pain but a significantly better cosmetic effect was achieved. The use of staplers in suturing the skin shortens the duration of the surgical procedure; although it is associated with an increased risk of wound dehiscence in obese women. RR 5.2 (95% CI 1.8–14.7) [30]. Similar results were obtained in a randomized cohort study involving 1147 women, in which sewing the skin with the staplers more than doubled the risk of postoperative wound dehiscence (RR 2.20; 95% CI 1.6–3.1) and contributed to its infection (wound infection or cellulitis) (RR 2.46; 95% CI 1.4–4.4) compared to the group of women who received intradermal sutures [28]. However, a 2018 study of women with grade III obesity did not confirm the above results [34].

#### 3.3.5. Subcutaneous Tissue Drainage

The prophylactic drainage of subcutaneous tissue aims to reduce the risk of formation of fluid reservoirs within a wound, which could disrupt its continuity or lead to its infection. The studies conducted by Al-Inany et al. [22] and Ramsey et al. [23] did not confirm the effectiveness of the prophylactic drainage of subcutaneous tissue. Alanis, in a retrospective cohort study, concluded that prophylactic drainage of subcutaneous tissue should be abandoned in women with massive obesity [24]. An American randomized cohort study showed that the drainage procedure significantly increases the risk of wound complications (OR 2.86; 95% CI 1.02–7.98), contributing to both its dehiscence and infection [27]. A study by Bindal and Munda based on obese women from India demonstrated that patients after a caesarean section with a drain had reduced rates of wound seroma, postoperative pain, and shorter hospital stays [35]. However, the authors did not observe any significant benefits of the drainage with regard to postoperative fever, superficial SSI, as well as wound breakdown [35]. A retrospective cohort study by Dias and colleagues [46] performed on severely obese women with a BMI of >40 kg/m^2^ showed no correlation between the use of a drain and SSI. A Japanese study analyzing two groups of women with a mean BMI of approx. 33 kg/m^2^, the first of which had staples and the second had subcuticular sutures and drains [25] found that the frequency of wound complications was significantly lower in women with a drain as compared to those with staples. The authors used four-channel Blake drains, which are made from soft fluted silicone with a wide surface area for drainage. This type of drain was demonstrated to be less painful in comparison to non-Blake drains [60]. On the other hand, Aziz Khalifa and colleagues reported a significant difference between obese women with a seroma drain after a caesarean section and women with no seroma drain (9.6% vs. 26.7%, respectively) and postoperative pain requiring analgesics [32]. Most of the available data regarding the usage of drains in post-caesarean women were obtained from studies performed on a low number of patients, which may have influenced the results. Thus, performing a meta-analysis may overcome the effect of a small analyzed population. To the best of our knowledge, no meta-analysis on the correlation between drainage use and wound complications in obese women after caesarean sections have been performed. The meta-analyses of Gates published in 2005 and 2013 [61] included studies analyzing women with both a normal BMI and overweight or obese women.

In the present study, we performed a meta-analysis concerning the comparisons of surgical complications after caesarean sections in obese women in terms of wound complications (including data on wound separation), infections (including data on SSI), as well as fevers.

**Figure 3:**
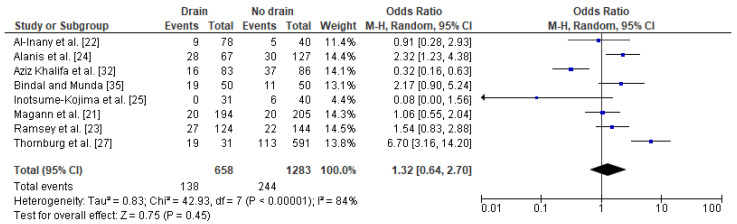
Forest plot for wound complications in the group of post-caesarean obese women with a drain and in the group of obese women without a drain. M.-H.—Mantel-Haenszel; CI—confidence interval; I^2^—heterogeneity; df—degrees of freedom.

In general, nine studies with a total number of 674 obese post caesarean women with a drain and 1718 obese women with no drain were included in the meta-analysis [21,22,23,24,25,27,32,35,46]. The impact of drainage on the risk of wound complications in obese women was conducted based on eight studies with a total number of 658 women with a drain and 1,283 obese women with no drain. Studies were highly heterogeneous (I^2^ 84%). The percentage of wound complications was slightly higher in obese women with a drain (21%) compared to obese women without a drain (19%). Since significant heterogeneity between studies was calculated, the REM method was used to calculate the pooled OR. The difference was not significant (OR 1.32; 95% CI 0.64–2.70, *p* = 0.45), which indicates that the use of drains does not increase the risk of wound complications in obese women after a caesarean section (Figure 3).

The results were stable after subsequent omitting each of the studies that was included. No publication bias was found for this analysis [Egger’s test (*p* = 0.577); Begg’s test (*p* = 0.458)].

**Figure 4:**
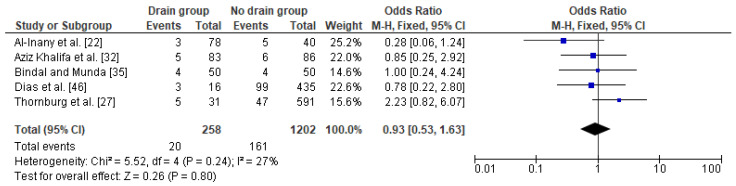
Forest plot for infections in the group of post-caesarean obese women with a drain and in the group of obese women without a drain. M.-H.—Mantel-Haenszel; CI—confidence interval; I^2^—heterogeneity; df—degrees of freedom.

The relation between drainage and infections (including SSI) after a caesarean section in obese women was based on five studies with a total number of 258 women with a drain and 1202 obese women with no drain. The percentage of wound complications was slightly lower in obese women with a drain (8%) as compared to obese women without a drain (13%). The heterogeneity between studies were at low level (I^2^ 27%) with no significance, therefore FEM method was used to calculate the pooled OR. The difference was not significant (OR 0.93; 95% CI 0.53–1.63, *p* = 0.80), which indicates no impact of drain usage on infections after a caesarean section in obese women (Figure 4). The results were stable during the sensitivity analysis; thus, the analysis is reliable. Again, no publication bias was observed [Egger’s test (*p* = 0.056); Begg’s test (*p* = 0.142)].

**Figure 5:**
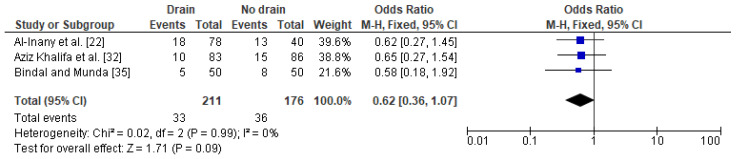
Forest plot for fever in the group of post-caesarean obese women with a drain and in the group of obese women without a drain. M.-H.—Mantel-Haenszel; CI—confidence interval; I^2^—heterogeneity; df—degrees of freedom.

In the case of the analysis of the impact of drainage on a fever after a caesarean section in obese women, three studies were included with a total number of 211 women with a drain and 176 obese women with no drain. The proportion of fevers was slightly lower in obese women with a drain (16%) as compared to obese women without a drain (20%). In this analysis, no heterogeneity between the studies was demonstrated (I^2^ 0%) and the FEM method was once again used to calculate the pooled OR. The difference was close to the border of significance (OR 0.62; 95% CI 0.36–1.07, *p* = 0.09) indicating that drain usage after a caesarean section may have some beneficial effect in the presence of a fever in obese women (Figure 5). The sensitivity analysis revealed that the results were stable and reliable and no publication bias was revealed [Egger’s test (*p* = 0.342); Begg’s test (*p* = 0.601)].

#### 3.3.6. Negative Pressure Wound Therapy Dressings (VAC Dressings)

Seven randomized trials concerning the effectiveness of vacuum dressings in the prevention of SSIs were carried out. Most of the studies indicated the ineffectiveness of the prophylactic use of the above therapy. The study conducted in women with a BMI of ≥30 kg/m^2^ did not show the negative pressure wound therapy (NPWT) dressing to significantly reduce the incidence of complications in the postoperative wound compared to standard wound care (4.9 vs. 6.9%; *p* = 0.71) [37]. Similar results were provided by the Hussama study that included 441 women with morbid obesity (BMI >40 kg/m^2^), where a closed vacuum dressing was used as compared to standard wound care, and also did not significantly reduce the incidence of complications in the skin (RR 0.9 [95% CI 0.5–1.4]) [43]. However, a Danish study from 2019 that encompassed 876 women with class I obesity or greater showed that SSIs were found in 4.6% in the NPWT dressing group, while in the control group there were 9.2% SSIs (RR 0.50; 95% CI 0.30–0.84); the number needed to treat 22; *p* = 0.007). The authors found that vacuum dressings used prophylactically in obese women significantly reduced the incidence of SSIs [43]. The results of the analysis are presented in Table 5.

**Figure 6:**
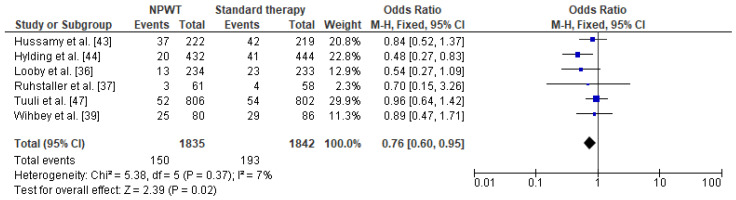
Forest plot for surgical site infection in the group of post-caesarean obese women with NPWT and in obese women with standard therapy. NPWT—negative pressure wound therapy; M.-H.—Mantel-Haenszel; CI—confidence interval; I^2^—heterogeneity; df—degrees of freedom.

Meta-analysis of the data regarding the frequency of SSI in obese women with NPWT in comparison to women with standard therapy was based on six studies with 1835 cases with NPWT therapy and 1842 patients with standard therapy. One of the studies was excluded because of incomplete data (absence of patient with standard therapy) [38]. This revealed that the prevalence of SSI was lower among obese women with NPWT than in women receiving standard wound therapy (8% vs. 10.5%, respectively). A significant reduction in the SSI odds ratio was observed in obese women with negative pressure wound therapy compared to obese women with standard therapy (OR = 0.76 95% CI 0.60–0.95, *p* = 0.02) (Figure 6). Very low level of heterogeneity between the studies was observed (I^2^ = 7%).

The results were stable after omitting one study at a time, i.e., the study by Hussamy et al. [43] first, then a study by Ruhstaller et al. [37], then a study by Wihbey et al. [39], and then a study by Tuuli et al. [47]. When the study by Hylding et al. [44] was omitted, the results changed and were not significant (OR = 0.84 95% CI 0.65–1.08, *p* = 0.17). Similarly, omitting the data by Looby et al. [36] gave insignificant results (OR = 0.79 95% CI 0.62–1.01, *p* = 0.06). Therefore the result of the meta-analysis should be treated with caution. The results of Egger’s and Begg’s tests showed no publication bias [Egger’s test (*p* = 0.456); Begg’s test (*p* = 0.348)].

## 4. Discussion

The use of a prophylactic dose of antibiotic in women undergoing a caesarean section reduces the risk of postoperative wound infections (RR 0.40; 95% CI 0.35–0.46, 82) and inflammation of the endometrial mucosa (endometritis) (RR 0.38; 95% CI 0.34–0.42, 83), as well as other complications resulting from infections (RR 0.31; 95% CI 0.20–0.49) [62]. The time of administration of a prophylactic dose of antibiotics also affects the frequency of infection. The studies show that the administration of an antibiotic before a surgical procedure reduces the percentage of endometritis by 41% compared to the intraoperative administration of an antibiotic (RR 0.59; 95% CI [95% CI] 0.37–0.94; I^2^ 0%), although such treatment does not reduce the incidence of postoperative wound infections (RR 0.71; 95% CI 0.44–1.14; I^2^ 0%) [63]. In clinical practice, the 1st-generation cephalosporin, Cefazolin, at a dose of 2 g, which has a spectrum of activity including Gram-positive bacteria and Escherichia coli, is used in the prophylaxis of perioperative caesarean sections. As evidenced by studies conducted among obese women who were not pregnant, cefazolin shows different pharmacokinetics in obese people, which means that its concentration is lower in adipose tissue. This phenomenon suggests that the prophylactic dose needs to be increased in such a group of obese patients [64]. The studies that we analyzed did not confirm the effectiveness of a higher dose of antibiotic in perioperative prophylaxis.

The suturing of subcutaneous tissue aims to reduce the risk of formation of fluid reservoirs. A meta-analysis of six studies showed that suturing reduces the risk of wound complications by 34% [65] in subcutaneous tissue that is thicker than 2 cm. A randomized study of 1082 women undergoing a caesarean section treated with antibiotic prophylaxis for more than 60 min from the skin incision, with the skin washed with an alcoholic chlorhexidine solution, and sutured subcutaneous tissue if its thickness was greater than 2 cm, as well as skin closed with sutures instead of staplers, showed a significant reduction in the risk of complications in the postoperative wound, regardless of the method of incision of the skin, the coexistence of obesity and diabetes, chorioamnionitis or the operator’s experience [11]. However, the studies analyzed by us and the meta-analysis on the method of incision of the skin demonstrated that a Pfannenstiel caesarean section reduces the risk of postoperative wound infections [66].

The prophylactic use of vacuum dressings in obese women is aimed at reducing the incidence of SSIs. As shown by a meta-analysis of seven studies, the prophylactic use of NPWT reduces the risk of SSIs (pooled RR 0.45; 95% CI 0.31, 0.66). Complex wound complications were significantly reduced in patients receiving prophylactic negative pressure wound therapy compared to standard dressings (9 studies: pooled RR 0.68; 95% CI 0.49, 0.94) [67]. Another meta-analysis conducted on 10 studies from 1966 to 2017 did not confirm that NPWT decreased wound complication (RR 0.97, 95 % CI 0.63–1.49) [68]. Moreover the most recent American study based on 1624 people also did not significantly confirm NPWT in reducing SSIs. [47] The analysis carried out by us, supplemented with the current 6 researches, showed, that NPWT reduces the risk of SSIs, nevertheless, the results should be treated with caution because the sensitivity analysis was not stable and no significance was observed after omitting the studies by Hylding et al. [44] and Looby et al. [36] no significance was observed.

The prophylactic use of subcutaneous drains to reduce SSIs remains controversial. The meta-analysis concerning the use of subcutaneous tissue drainage after surgical procedures did not show a reduction in incidence of complications in the postoperative wounds; hence, it is unjustified in everyday surgical practice [69], as well as in caesarean sections and other surgical procedures performed in obese patients [70], which was confirmed in our meta-analysis.

The strength of our study is the meta-analysis of the research on the prophylactic use of a subcutaneous drain during a caesarean section procedure in obese women. It should be noted that no such analysis has been carried out to date. In addition, current research on the NPWT confirms that SSIs are significantly infrequent in obese women with NPWT, however, further analysis should provide more studies to confirm stable and reliable results.

The relatively small number of studies and significant variability in outcome reporting are important limitations of our study. All analyzed studies by us were in English language, which also might be a limitation of the study. Moreover, a considerable amount of research concerning the issue of caesarean sections includes retrospective studies that do not comprise clinical indications for a surgical procedure and do not refer to data regarding the duration of amniotic fluid leakage, the presence of uterine contractions, and a history of diabetes. Furthermore, the authors of some studies did not differentiate between caesarean sections in schedule and emergency ones [10,26,34,41]. The heterogeneity of the studies included in the review also constitutes a limitation of the study.

## 5. Conclusions

This paper studies the optimal management of a caesarean section wound including a transverse incision of the skin. NPWT reduces risk of surgical site infection. On the other hand subcutaneous drain does not reduce the risk of wound complications, wound infections, and fever in obese women after a caesarean section. The optimal dose of antibiotics in perioperative prophylaxis is still under investigation; therefore, it is advisable to conduct further multicenter studies on the caesarean section procedure in obese women.

## Figures and Tables

**Table 1:** Characteristics of studies included in the systematic review and meta-analysis.

Study	Date	Country	Study Design	*N*	Exclusion Criteria	Definition of Obesity	Primary Outcomes
Magann et al., 2002 * [21]	1998–2001	Australia	Prospective randomized study	964	- Declined participation in investigation, - Emergency caesarean section without consent to participate in the study	At least 2 cm of subcutaneous fat tissue on admission to delivery	Risk of wound disruption after caesarean delivery
Al-Inany et al., 2002 * [22]	1999–2000	Egypt	Prospective controlled clinical study	118	- Prolonged premature rupture of membranes, - Prolonged labor, - Long preoperative hospitalization, - Malignancy, - Diabetes mellitus, - Chronic lung disease	BMI > 32 kg/m^2^ subcutaneous fat of at least 2 cm	The incidence of wound breakdown in both groups together with the rate of hematoma formation and occurrence of fever
Ramsey et al., 2005 * [23]	2001–2004	USA	Randomized study	280	- Inability to obtain informed consent, - Moribund caesarean delivery required, - No plan for follow-up	BMI > 30 kg/m^2^ on admission	Composite wound morbidity rate, defined as any of the following noted during the post-hospital discharge wound follow-up assessments: subcutaneous dehiscence (1 cm), seroma, hematoma, abscess, or fascial dehiscence
Alanis et al., 2010 * [24]	2005–2009	USA	Retrospective cohort study	194	- Maternal death	Pre-delivery BMI ≥ 50 kg/m^2^	Wound complications (wound disruption, cellulitis, NOT superficial wound
Inotsume-Kojima et al., 2011 * [25]	2006–2009	Japan	Retrospective cohort study	71	- Informed consent for the new surgical method was not obtained preoperatively, - A Pfannenstiel incision was made, - Surgery revealed malignancy, - Subcutaneous fat thickness was <2 cm, - Hospitalization before surgery was longer than 24 h, - Surgery was performed in response to premature labor, - Patient was not a native of Japan	BMI ≥ 28 kg/m^2^ at the time of admission	Wound complications
McLean et al., 2011 [26]	1998–2005	USA	Retrospective cohort study	242	Incomplete medical reports	BMI ≥ 30 kg/m^2^ at delivery	Type of skin incision and partial or complete wound separation
Thornburg et al., 2012 * [27]	1994–2008	USA	Retrospective cohort study	623	- Prior caesarean delivery, - Skin incision other than vertical or low transverse, - Missing prepregnancy height or weight, or unavailable follow-up data	BMI ≥ 35 kg/m^2^ at delivery	Presence of any wound complication defined as a wound separation, including both spontaneous and indicated as resulting from seroma formation or wound infection/cellulitis
Subramanian et al., 2014 [28]	2009–2010	USA	Retrospective cohort study	340	- Primary wound infections (purulent drainage, cellulitis, and/or abscess requiring antibiotics or surgical treatment)	BMI ≥ 30 kg/m^2^ at delivery	Risk factors for non-infectious wound disruption following a caesarean delivery
Stamilio et al., 2014 [29]	2008–2010	USA	Retrospective cohort study	585	- Emergency surgery - Human immunodeficiency virus infection, - Chronic corticosteroid therapy or other immunosuppressive therapy, - General anesthesia, - Diagnosis of extrauterine infection	BMI ≥ 30 kg/m^2^ at delivery	Composite of wound infection and endometritis
Conner et al., 2014 [10]	2004–2008	USA	Retrospective cohort study	2444	- No completed follow-up data	BMI ≥ 30 kg/m^2^ at delivery	A wound complication defined as the occurrence of a wound seroma, hematoma, separation, dehiscence or infection from the time of delivery to 6 weeks postoperative
Ibrahim et al., 2014 [30]	March 2012–August 2012	Egypt	Retrospective cohort study	130	- Infection (e.g., chorioamnionitis, pyelonephritis or chest infection), intraoperative events predispose to perioperative infection (e.g., bowel injury, - Operative time more than 90 min, - Major blood loss (hemoglobin less than 10 g/dL), - Pre-eclampsia, diabetes mellitus or rupture of membranes for more than 12 h, - Immunosuppressive drugs, - non-Pfannenstiel incision, - nonelective caesarean section, - BMI <30 kg/m^2^	BMI ≥ 30 kg/m^2^ at delivery	Superficial incisional surgical site infection (SSI)
Smid et al., 2015 [31]	1999–2002	USA	Retrospective cohort study	38,299	- Incomplete demographic, exposure, or outcome data	BMI ≥ 30–45 kg/m^2^ extremely obese BMI ≥ 45 kg/m^2^ at delivery	A wound complication composite of a wound infection, endometritis, a wound opening, a seroma/hematoma, and hospital readmission
Khalifa et al., 2015 * [32]	2012–2013	Egypt	Randomized controlled trial	169	- Major intraoperative complications (bowel or urinary tract injuries, massive blood loss, transfusion)	BMI ≥ 30 kg/m^2^	The rate of superficial surgical site infection
Sutton et al., 2016 [33]	2010–2013	USA	Retrospective cohort study	421	- Prenatally diagnosed fetal anomalies, - Planned caesarean hysterectomies	BMI ≥ 40 kg/m^2^ at the time of delivery	A wound composite (cellulitis, abscess, hematoma, seroma, or dehiscence)
Zaki et al., 2016 [34]	2006–2011	USA	Retrospective cohort study	1147	- Women with fascial dehiscence, - Multipregnancy, - Second pregnancy	Pre pregnancy BMI ≥ 30 kg/m^2^	A composite of wound disruption or infection occurring within 6 weeks postpartum
Bindal & Munda, 2017 * [35]	2015–2016	India	Retrospective cohort study	100	- Major intraoperative complications (bowel or urinary tract injuries, massive blood loss, and transfusion)	BMI ≥ 30 kg/m^2^ at the time of caesarean section	The rate of superficial surgical site infection defined as the presence of wound discharge that yielded a positive result on bacteriological culture
Looby et al., 2017 ** [36]	2007–2014	USA	Retrospective cohort study	467	- Vaginal delivery - BMI less than 40 kg/m^2^, - Loss of follow-up, - Multiple deliveries - Use of nonpunch through (NPT) devices prior to official start date	BMI ≥ 40 kg/m^2^	A surgical site infection (SSI), within 30 days of surgery
Rusthaller et al., 2017 ** [37]	2014–2016	USA	Retrospective cohort study	136	- Initiation of prenatal care after 23 weeks (no early BMI), - Chronic steroid use, - Pregestational diabetes, treatment for an active malignancy, - Allergy to silver (contraindication to Prevena negative pressure wound therapy device (NPWT), - Scheduled caesarean section, - Planned vertical skin incision	BMI ≥ 30 kg/m^2^ at the first prenatal visit <22 weeks	A composite of wound morbidity at 4 weeks postpartum including a surgical site infection (SSI) and/or wound opening
Temming et al., 2017 [11]	2011–2015	USA	Retrospective cohort study	1082	- Allergy to chlorhexidine, alcohol, iodine or shellfish, - Skin infection near the operative site, - Without follow-up after discharge	BMI ≥ 30 kg/m^2^	A composite of wound complications, including surgical site infection (SSI), cellulitis, seroma, hematoma, and separation within 30 days
Searle et al., 2017 ** [38]	2012–2016	Ireland, England	Retrospective cohort study	399	- Patient BMI < 35 kg/m^2^ or patient BMI missing; - Missing data in follow-up time (time between procedure date and follow-up date) less than seven days or missing	BMI ≥ 35 kg/m^2^ at the time of caesarean section	Postoperative wound complications including surgical site infection (SSI)
Wihbey et al., 2018 ** [39]	2015–2017	USA	Retrospective cohort study	166	- Under 18 years old, - Did not speak English, - Had an allergy to silver or adhesive products, - Skin incision that would not fit the device or standard dressing (e.g., “T” skin incision)	BMI ≥ 35 kg/m^2^	A superficial surgical site infection, an infection involving only the skin or subcutaneous tissue occurring within 30 days of surgery with at least one of the following: purulent drainage from the wound or organism identified by culture or wound deliberately opened by the surgeon
Zaki et al., 2018 [40]	205–2016	USA	Retrospective cohort study	242	- Hypersensitivity to staples, - Potential immunosuppression including infection with human immunodeficiency virus, chronic steroid use or active lupus	BMI ≥ 40 kg/m^2^	A composite wound complication defined as a superficial or deep separation and infection occurring up to 6 weeks following delivery
Alalfy et al., 2018 [41]	From June 2017–December 2017	Egypt	Retrospective cohort study	397	- BMI <30 kg/m^2^ - Previous caesarean section - Medical disorders diabetes mellitus, hypertension with pregnancy	BMI ≥ 30 kg/m^2^	Wound outcome results regarding postoperative wound complications compared to two widely implemented techniques in subcutaneous tissue closure (interrupted versus continuous methods)
Marrs et al., 2019 [42]	2013–2017	USA	Retrospective cohort study	91	- Rupture of membranes for more than 18 h, - Clinical chorioamnionitis at the time of delivery, - Subsequent vaginal delivery, - Participants enrolled in other trials, - Women with strong indications for a certain skin incision type (i.e., placenta accreta necessitating caesarean hysterectomy)	BMI ≥ 40 kg/m^2^	A composite wound complication that included any of the following: surgical site infection (SSI), cellulitis, seroma/hematoma, or separation up to 6 weeks postpartum
Hussamy et al., 2019 ** [43]	2015–2016	USA	Retrospective cohort study	441	- Anticoagulation therapy, - Human immunodeficiency virus infection, - Silver or acrylic allergy	BMI ≥ 40 kg/m^2^ measured within 2 weeks of admission for delivery	A wound complication defined as a wound disruption or wound infection (including cellulitis)
Hyldig et al., 2019 ** [44]	2013–2016	Denmark	Retrospective cohort study	876	- Vaginal delivery, - Missing data	Pre-pregnancy BMI ≥ 30 kg/m^2^	A surgical site infection was defined as a surgical site infection requiring antibiotic treatment within the first 30 days after the caesarean section
Connery et al., 2019 [45]	2013–2016	USA	Retrospective cohort study	657	- Patients did not receive routine prophylactic dose of antibiotics in the operating room, - Skin incisions other than Pfannenstiel, - Uterine incisions other than low transverse, - Patients with known or discovered allergies to silver or nylon	BMI ≥ 40 kg/m^2^	A superficial surgical site infection at any time within the first 6 weeks after caesarean delivery
Dias et al., 2019 * [46]	2011–2015	Scotland	Retrospective cohort study	453	-missing data	BMI > 40 kg/m	Maternal and surgical predictors of surgical site infection
Tuuli et al., 2020 ** [47]	2017–2019	USA	Retrospective cohort study	1624	- Postoperative follow-up not available, - Contraindication to negative pressure use (pre-existing infection at the incision site), - Bleeding disorder, - Therapeutic anticoagulation, - Allergy to silicone or adhesive tape	BMI ≥ 30 kg/m^2^ at or beyond 23 weeks of gestation	A superficial or deep surgical site infection

* Studies evaluate subcutaneous drainage in obese women after a caesarean section. ** Studies evaluate prophylactic negative pressure wound therapy in obese women after a caesarean section.

**Table 2:** Characteristics of studies evaluating prophylactic antibiotics included in the systematic review.

Study	Country	Study Design	*N*	BMI of Participants	Primary Outcomes	Dose of Antibiotics	Results
Pevzner et al., 2011 [48]	USA	Prospective study	29	BMI < 30 kg/m^2^ (*n* = 10) BMI 30–39.9 kg/m^2^ (*n* = 10) BMI ≥ 40 kg/m^2^ (*n* = 9)	Cefazolin concentration in adipose tissue and surgical site infection	2 g of cefazolin 30–60 min before skin incision	No significant difference in cefazolin concentration observed in mean closure adipose, myometrial or serum specimens across the BMI categories
Stitely et al., 2013 [49]	New Zealand	Retrospective cohort study	20	BMI ≥ 35 kg/m^2^	Tissue concentration of antibiotics	Cefazolin 2 g vs. 4 g i.v.	Mean cefazolin plasma, umbilical cord, and myometrial concentrations significantly higher in the 4 g treatment group (*p* < 0.05)
Ahmadzia et al., 2015 [50]	USA	Retrospective cohort study	335	>250 pounds	Incidence of surgical site infections, (superficial, deep, and organ/space—i.e., endometritis infections)	Cefazolin 2 g vs. cefazolin 3 g	No difference in surgical site infection among women who received 2 g compared with 3 g cefazolin
Young et al., 2015 [51]	USA	Double-blind randomized controlled trial	28	A pregnancy BMI ≥ 30 kg/m^2^	Cefazolin Concentrations in maternal plasma, umbilical cord blood, and maternal adipose tissue	2 g or 3 g cefazolin concentrations within 30 min of skin incision	Cefazolin concentrations in plasma and adipose tissue were related to both dose and body mass index. No difference between 2 and 3 g cefazolin doses to maintain adipose tissue concentrations above the minimum inhibitory concentration
Swank et al., 2015 [52]	USA	Prospective control study	29	BMI ≥ 30 kg/m^2^	Cefazolin concentration in tissue	2 g or 3 g cefazolin concentrations within 30–60 min of skin incision	Higher adipose concentrations of cefazolin were observed after the administration of an increased prophylactic dose
Maggio et al., 2015 [53]	USA	Double-blind randomized controlled trial	57	BMI ≥ 30 kg/m^2^	Adipose tissue cefazolin concentration	2 g vs. 3 g cefazolin	Prophylaxis with 3 g of cefazolin did not significantly increase adipose tissue concentration
Lilico et al., 2016 [54]	Canada	Prospective control study	6	BMI ≥ 35 kg/m^2^	Cefazolin concentration in tissue	25 mg/kg of cefazolin	Obese patients required a higher dose of cefazolin
Groff et al., 2017 [55]	USA	Prospective controlled study	8	BMI ≥ 30 kg/m^2^	Prevention of wound infection	2 g of cefazolin	No difference between groups in total and free cefazolin concentrations
Gupper et al., 2017 [56]	USA	Analysis of 3 retrospective controlled studies	67	BMI ≥ 30 kg/m^2^	Cefazolin adipose tissue concentration	2 g or 3 g cefazolin concentrations within 30–60 min of skin incision	A 2 g dose of cefazolin had a high probability of providing adipose tissue concentrations above the target pathogens’ MIC for overweight and obese females
Kram et al., 2017 [57]	USA	Prospective controlled study	84	BMI ≥ 30 kg/m^2^	Cefazolin blood and adipose tissue concentration	2 g and 3 g of cefazolin for body weights <120 kg and ≥120 kg	Dosage groups did not differ in cefazolin concentration (median [interquartile range]) in adipose tissue following skin incision, and in adipose tissue before skin closure. Mean concentrations were significantly lower than the MIC of 8 mg/g (*P* < 0.03) in both groups
Eley et al., 2020 [58]	Australia	Prospective study	12	BMI ≥ 35 kg/m^2^	Plasma and interstitial fluid pharmacokinetics of intravenous cefazolin	2 g of cefazolin i.v.	Wound closure did not occur within 2 h; redosing is suggested following either a 2 or 3 g initial bolus

**Table 3:** Results of studies that included a prophylactic dose of cefazolin.

Study	*N*	BMI of Participants	Primary Outcomes	Dose of Antibiotics	Results
Pevzner et al., [48]	29	BMI < 30 kg/m^2^ (*n* = 10) BMI 30–39.9 kg/ m^2^ (*n* = 10) BMI ≥ 40 kg/m^2^ (*n* = 9)	Cefazolin concentration in adipose tissue and surgical site infection	2 g of cefazolin 30–60 min before the skin incision	No significant difference in cefazolin concentration was observed in mean closure adipose, myometrial, or serum specimens across the BMI categories
Stitely et al., [49]	20	BMI ≥ 35 kg/m^2^	Tissue concentration of antibiotics	Cefazolin 2 g vs. 4 g i.v.	The mean cefazolin plasma, umbilical cord, and myometrial concentrations were significantly higher in the 4 g treatment group (*p* < 0.05)
Ahmadzia et al., [50]	335	>250 pounds	Incidence of surgical site infections, (superficial, deep, and organ/space—i.e., endometritis) infections)	Cefazolin 2 g vs. cefazolin 3 g	No difference in surgical site infection among women who received 2 g compared with 3 g cefazolin
Young et al., [51]	28	Pregnancy BMI ≥ 30 kg/m^2^	Cefazolin concentrations in maternal plasma, umbilical cord blood, and maternal adipose tissue	2 g or 3 g within 30 min of the skin incision	Cefazolin concentrations in plasma and adipose tissue are related to both the dose and the body mass index. No difference between 2 g and 3 g cefazolin doses to maintain adipose tissue concentrations above the minimum inhibitory concentration
Swank et al., [52]	29	BMI ≥ 30 kg/m^2^	Cefazolin concentration in tissue	2 g or 3 g within 30–60 min of the skin incision	Higher adipose concentrations of cefazolin were observed after the administration of an increased prophylactic dose
Maggio et al., [53]	57	BMI ≥ 30 kg/m^2^	Adipose tissue cefazolin concentration measured by high pressure liquid chromatography	2 g vs. 3 g cefazolin	Prophylaxis with 3 g of cefazolin did not significantly increase adipose tissue concentration
Lilico et al., [54]	6	BMI ≥ 35 kg/m^2^	Cefazolin concentration in tissue	25 mg/kg of cefazolin	Obese patients need a higher dose of cefazolin
Groff et al., [55]	8	BMI ≥ 30 kg/m^2^	Prevention of wound infection	2 g of cefazolin	No difference between groups in total and free cefazolin concentrations
Gupper et al., [56]	67	BMI ≥ 30 kg/m^2^	Cefazolin adipose tissue concentration	2 g or 3 g within 30–60 min of skin incision	2 g dose has a high probability of providing adipose tissue concentrations above the target pathogens’ MIC for overweight and obese females
Kram et al., [57]	84	BMI ≥30 kg/m^2^	Cefazolin blood and adipose tissue concentration	2 g and 3 g of cefazolin for body weights of <120 kg and ≥120 kg	Dosage groups did not differ in cefazolin concentration (median [interquartile range]) in adipose tissue following the skin incision, and in adipose tissue before the skin closure. Mean concentrations were significantly lower than the MIC of 8 mg/g (*P* < 0.03) in both groups
Eley et al., [58]	12	BMI ≥ 35 kg/m^2^	plasma and interstitial fluid pharmacokinetics of intravenous cefazolin	2 g of cefazolin i.v.	Wound closure did not occur within 2 h; redosing is suggested following either a 2 or 3 g initial bolus

BMI—body mass index.

**Table 4:** Types of skin incisions and wound complications.

Study	*N*	BMI (kg/m^2^)	Wound Complications		Results
			*N*	OR	
Alanis et al. [24]	194	≥50	V 39/104 T 19/90	2.24 (1.18–4.27)	Vertical abdominal incisions were associated with wound complications
McLean et al. [26]	242	≥30	V 5/25 T 22/213	2.17 (0.74–6.36)	Type of skin incision does not appear to be associated with wound complications in obese patients
Tornburg et al. [27]	623	≥35	V 16/35 T 68/588	6.44 (3.16–13.12)	Vertical skin incisions are associated with an increased risk of wound complications
Smid et al. [31]	2411	>45	V 113/669 T 17/224	2.47 (1.45–4.22)	Vertical skin incisions are associated with an increased risk of wound complications
Sutton et al. [33]	421	≥40	V 15/57 T 54/364	2.05 (1.06–3.95)	Vertical incisions are associated with more wound complications
Marrs et al. [42]	91	≥40	V 8/38 T 8/43	1.17 (0.39–3.49)	Pfannenstiel skin incisions were associated with lower wound complications

V—vertical skin incision, T—transverse skin incision, N—number of cases, OR—odds ratio, BMI—body mass index.

**Table 5:** Results of studies included in the review and meta-analysis describing the role of prophylactic negative pressure therapy in surgical site infections.

Study	*N*	BMI (kg/m^2^)	Surgical Site Infection (SSI)		Results
			*N*	OR	
Looby et al. [36]	467	≥40	NPWT 13/234 SD 23/233	0.45 (0.22–0.95)	NPT after caesarean delivery in women with a BMI of ≥40 kg/m^2^ is an efficacious method to reduce the incidence of postoperative wound infections
Ruhstaller et al. [37]	119	≥ 30	NPWT 3/61 SD 4/58	0.70 (0.15–3.26)	Routine clinical use of a NPWT system after a caesarean delivery did not result in a significant reduction in wound morbidity over standard wound complications
Searle et al. [38]	399	≥35	NPWT 36/399	-	Use of NPWT on closed surgical incisions may be associated with low incidence of SSI
Wihbey et al. [39]	166	≥35	NPWT 25/80 SD 29/86	0.89 (0.47–1.71)	There were no differences in the occurrence of composite wound complications between women using prophylactic NPWT
Hussamy et al. [43]	441	≥40	NPWT 37/222 SD 42/219	0.84 (0.52–1.37)	A NPWT device compared with a standard dressing did not significantly lower the wound complication rate in morbidly obese women undergoing caesarean delivery
Hylding et al. [44]	876	≥ 30	NPWT 20/432 SD 41/444	0.5 (0.30–0.84)	Prophylactic use of incisional NPWT reduced the risk of surgical site infections
Tuuli et al. [47]	1624	≥ 30	NPWT 52/806 SD 54/802	0.95 (0.66–1.37)	Prophylactic NPWT compared with a standard wound dressing did not significantly reduce the risk of surgical-site infection

NPWT—negative pressure wound therapy, SD—standard therapy, N—number of cases, OR—odds ratio, BMI—body mass index.

## Supplementary Materials

The following are available online at https://www.mdpi.com/2077-0383/10/4/675/s1. Table S1: Jadad’s scale.
