# Supplementary material for: A Systematic Review and Meta-Analysis of Wound Complications after a Caesarean Section in Obese Women
Source: J Clin Med. 2021 Feb 10;10(4):675. doi: 10.3390/jcm10040675 (PMC7916387; doi:10.3390/jcm10040675)
Supplement: Supplementary file 1 [file jcm-10-00675-s001.pdf]

Table Supplementary 1. Jadad's scale

Author	Year	Country	Was a study described as random?	Was a study described as double blinded?	Was there a description of exclusion criteria?	Was the randomization scheme described and appropriate?	Was the method of double blinding appropriate?
Magann et al.	2002	Australia	+	-	+	+	-
Al-Inany et al.	2002	Egypt	+	-	+	+	-
Ramsey et al.	2005	USA	+	-	+	+	-
Alanis et al.	2010	USA	+	-	-	-	-
Inotsume-Kojima et al.	2011	Japan	+	-	+	+	-
McLean et al.	2011	USA	+	-	-	-	-
Pevzner et al.	2011	USA	-	-	+	-	-
Thornburg et al.	2012	USA	+	-	+	+	-
Stitely et al.	2013	New Zealand	+	-	+	+	-
Subramaniam et al.	2014	USA	+	-	+	-	-

Stamilio et al.	2014	USA	+	-	+	-	-
Conner et al.	2014	USA	+	-	-	-	-
Ibrahim et al.	2014	Egypt	+	-	+	+	-
Khalifa et al.	2015	Egypt	+	-	+	+	-
Ahmadzia et al.	2015	USA	-	-	+	-	-
Young et al.	2015	USA	+	+	+	+	+
Swank et al.	2015	USA	+	-	+	-	-
Maggio et al.	2015	USA	+	+	+	+	+
Smid et al.	2015	USA	+	-	+	-	-
Zaki et al.	2016	USA	+	-	+	-	-
Sutton et al.	2016	USA	+	-	+	-	-
Lilico et al.	2016	Canada	-	-	+	-	-
Bindal & Munda	2017	India	+	-	+	+	-
Temming et al.	2017	USA	+	-	+	+	-

Ruhstaller et al.	2017	USA	+	-	+	+	-
Searle et al.	2017	Ireland England	+	-	-	-	-
Looby et al.	2017	USA	+	-	+	+	-
Groff et al.	2017	USA	+	-	+	+	-
Gupper et al.	2017	USA	+	-	+	+	-
Kram et al.	2017	USA	+	-	+	+	-
Wihbey et al.	2018	USA	+	-	-	+	-
Hussamy et al.	2019	USA	+	-	+	+	-
Zaki et al.	2018	USA	+	-	+	+	-
Alalfy et al.	2018	Egypt	+	-	+	+	-
Marrs et al.	2019	USA	+	-	+	+	-
Hylding et al.	2019	Denmark	+	-	+	+	-
Connery et al.	2019	USA	+	+	+	+	+
Dias et al.	2019	Scotland	-	-	+	-	-

Tuuli et al.	2020	USA	+	-	+	+	-
Eley et al.	2020	Australia	-	-	+	-	-
